# Language Tasks and the Network Control Role of the Left Inferior Frontal Gyrus

**DOI:** 10.1523/ENEURO.0382-20.2021

**Published:** 2021-09-08

**Authors:** John D. Medaglia, Denise Y. Harvey, Apoorva S. Kelkar, Jared P. Zimmerman, Joely A. Mass, Danielle S. Bassett, Roy H. Hamilton

**Affiliations:** 1 Department of Psychology, Drexel University, Philadelphia, PA 19104; 2 Department of Neurology, Drexel University, Philadelphia, PA 19104; 3 Department of Neurology, University of Pennsylvania, Philadelphia, PA 19104; 4 Sidney Kimmel Medical College, Thomas Jefferson University, Philadelphia, PA 19107; 5 Department of Psychiatry, University of Pennsylvania, Philadelphia, PA 19104; 6 Department of Bioengineering, University of Pennsylvania, Philadelphia, PA 19104; 7 Department of Physics and Astronomy, University of Pennsylvania, Philadelphia, PA19104; 8 Department of Electrical and Systems Engineering, University of Pennsylvania, Philadelphia, PA 19104; 9 Santa Fe Institute, Santa Fe, NM 87501

## Abstract

Recent work has combined cognitive neuroscience and control theory to make predictions about cognitive control functions. Here, we test a link between whole-brain theories of semantics and the role of the left inferior frontal gyrus (LIFG) in controlled language performance using network control theory (NCT), a branch of systems engineering. Specifically, we examined whether two properties of node controllability, boundary and modal controllability, were linked to semantic selection and retrieval on sentence completion and verb generation tasks. We tested whether the controllability of the left IFG moderated language selection and retrieval costs and the effects of continuous θ burst stimulation (cTBS), an inhibitory form of transcranial magnetic stimulation (TMS) on behavior in 41 human subjects (25 active, 16 sham). We predicted that boundary controllability, a measure of the theoretical ability of a node to integrate and segregate brain networks, would be linked to word selection in the contextually-rich sentence completion task. In contrast, we expected that modal controllability, a measure of the theoretical ability of a node to drive the brain into specifically hard-to-reach states, would be linked to retrieval on the low-context verb generation task. Boundary controllability was linked to selection and to the ability of TMS to reduce response latencies on the sentence completion task. In contrast, modal controllability was not linked to performance on the tasks or TMS effects. Overall, our results suggest a link between the network integrating role of the LIFG and selection and the overall semantic demands of sentence completion.

## Significance Statement

Our understanding of language systems and responses to neural stimulation is incomplete. Here, we demonstrate that the effects of neuromodulation (transcranial magnetic stimulation; TMS) on verbal language production are linked to the role of the left inferior frontal gyrus (LIFG) in mediating communication across white matter anatomic networks. We replicate prior findings in weighted anatomic networks, and further identify a link between the role of the LIFG in word selection demands. These findings provide a critical basis to reconcile local and whole brain models of language in the brain.

## Introduction

Effective language production requires cognitive control: the mental processes that support flexible, contextually driven thought and action (Snyder et al., 2011). In contrast to cognitive control tasks that require inhibition of single prepotent exemplars, language tasks are frequently underdetermined, multiple responses might be appropriate (Snyder et al., 2014). Fluent language requires the ability to meet word retrieval (recalling task-appropriate words) and selection (selecting a subset of retrieved words to speak) demands when speaking. However, selection and retrieval demands vary based on the nature of specific tasks, sentence structures, and word combinations. In some cases, retrieving and selecting words is difficult and accompanied by a sense of subjective effort, such as when the appropriate words do not readily come to mind or when many appropriate, alternative words compete for selection.

Cognitive control facilitates language production by activating the relevant representations and resolving competition among the activated representations (Badre and Wagner, 2007). Broca’s area, part of the left inferior frontal gyrus (LIFG), has been linked to retrieval and selection via interactions with temporal lobe regions that mediate semantic knowledge (Anwander et al., 2007; Harvey et al., 2013). However, debates about the neuroanatomical basis of cognitive control in language remain. It is unclear whether retrieval and selection localize to the same region or different subdivisions within the LIFG, reflecting the same or different mechanistic roles (Souza et al., 2009; Fedorenko et al., 2012). Conflicting accounts have asserted that the LIFG is implicated only in selecting a single response from among competing alternatives (Thompson-Schill et al., 1997; Botvinick et al., 2001), only in effortful retrieval of responses from semantic memory (Wagner et al., 2001; Martin and Cheng, 2006), or in both retrieval and selection through different neural substrates within the LIFG (Badre and Wagner, 2007) or through shared neural substrates with different, albeit not unrelated, mechanisms (Snyder et al., 2011).

Whereas localizationist accounts focus on the role of LIFG and left temporal regions in language production, the role of domain general and specific cognitive control and their representation in brain networks remains a persistent issue (Crinion et al., 2006; Fedorenko and Thompson-Schill, 2014; Diachek et al., 2019; Ryskin et al., 2020). Moreover, the role of distributed brain networks in semantic processing is an open question, with some accounts contending that the entire brain contributes to semantic representation (Patterson et al., 2007; Huth et al., 2012; Çukur et al., 2013; Bruffaerts et al., 2019; Shahdloo et al., 2020). The focus of the current study is on multiple network roles the IFG may play based on its anatomic position in brain networks. However, the extent to which these roles relate to selection and retrieval demands in language production has not been established.

To investigate the network roles of the LIFG relevant to language demands, we applied an emerging area of engineering called network control theory (NCT; Liu et al., 2011) to brain networks. NCT evaluates the nature and costs of control strategies in networks used to achieve target states. Network controllability is the ability of parts of a network (e.g., specific regions in the brain) to guide the network to target states. In a broad sense, cognitive control in the language domain is a special case of a network control problem for the brain (Medaglia, 2019): how does the brain achieve the neural states necessary to produce context-appropriate responses? Since the first theoretical network controllability analyses in large scale diffusion MRI networks (Gu et al., 2015), NCT has been used to characterize the energy required to integrate or segregate network activity (Betzel et al., 2016; Gu et al., 2017; Tang et al., 2017; Wu-Yan et al., 2020), identify correlates of cognitive function in and out of the executive domain (Kenett et al., 2018a,b; Cornblath et al., 2019; Lee et al., 2020), and predict or correlate the effects of brain stimulation on the brain and behavior (Medaglia et al., 2018a; Khambhati et al., 2019; Stiso et al., 2019; Beynel et al., 2020).

Building on our previous study (Medaglia et al., 2018a), the current study specifically investigated (1) retrieval and selection demands in verbal language production (2) task-level differences in sentence completion and verb generation using weighted anatomic networks. We used NCT to compute the controllability of the LIFG within distributed brain networks. In NCT, a brain network can be represented as graphs that comprise nodes (e.g., brain regions) and edges (e.g., anatomic connections between regions; Gu et al., 2015; Medaglia et al., 2018a; Patankar et al., 2020). We asked whether LIFG network controllability influenced language performance variability related to task-level and item-level differences in demands. We expected that LIFG controllability would predict performance variability during sentence completion and verb generation tasks. We hypothesized that boundary controllability, the theoretical ability of a region to drive networks into integrated or segregated states, would be positively related to sentence completion performance, facilitating semantic processes that rely on multiple networks. For both tasks, we predicted that higher boundary controllability would be associated with reduced selection costs before administering TMS. In contrast, we expected that modal controllability, the ability of a region to easily drive the brain into difficult-to-reach states, would be more related to the decontextualized, single-exemplar retrieval demands required in a verb generation task, since that task requires subjects to generate a single word in response to a cue, where there is no contextual information/meaning (unlike a sentence). Regarding neuromodulation effects, we expected that boundary controllability would moderate transcranial magnetic stimulation (TMS) effects on overall sentence completion performance and selection demands. In contrast, we expected that TMS effects would interact with retrieval demands in verb generation and would be moderated by modal controllability. These relationships would provide further evidence of demand-controllability associations within the LIFG.

## Materials and Methods

### Subjects

Forty-one healthy individuals (mean age = 25.3, SD = 5.9, 23 female) were scanned on a 3T Prisma scanner at the University of Pennsylvania in the present study. There were 16 subjects (age: 25.67, SD = 7.03) in the sham group and 25 subjects (age: 25.20, SD = 4.9) in the active group. Our previous study included *n* = 32 (12 sham, 20 active) subjects (Medaglia et al., 2018a). From the previous *n* = 32 sample, two left-handed subjects (from the active group) and two subjects with English as a Second Language (from the sham group) were excluded for the current study, leaving 28 subjects from the previous study included in the current study. The 13 new subjects were right-handed native English speakers with seven subjects in the sham group and six subjects in the active group. All procedures were approved in a convened review by the University of Pennsylvania’s Institutional Review Board and were conducted in accordance with the guidelines of the Institutional Review Board/Human Subjects Committee, University of Pennsylvania. All participants volunteered with informed consent in writing before data collection.

### Overview of methods

Network controllability characterizes the theoretical ability of a node in a network (e.g., a region in the brain) to drive the state of network activity Liu et al. (2011). Here, we built on our previous work linking boundary controllability to performance on open-ended language tasks and modal controllability to closed-ended language tasks Medaglia et al. (2018a). Specifically, the current study focused on task-level differences between two open-ended tasks, sentence completion and verb generation, and two dimensions of language demands, selection and retrieval (Snyder and Munakata, 2008; Snyder et al., 2014). Sentence completion task stimuli contain additional grammatical structure and contextual semantics than verb generation task stimuli. Intuitively, we expected that these processing demands would rely on multiple brain networks, and the theoretical role of the LIFG in mediating among networks could be measured with boundary controllability. In contrast, verb generation task stimuli might place greater demands on the LIFG when subjects must obtain associations in the absence of additional task structure or cues. We expected that if these demands are reflected in the LIFG’s role in achieving difficult-to-reach states (i.e., specifically states of activation that are otherwise difficult to activate in the network), we would find a relationship between performance on verb generation and modal controllability. In addition, both tasks stratified selection and retrieval demands at the item level, and we expected that the effects of these demands on performance would be moderated by boundary and modal controllability, respectively. We anticipated that boundary controllability would facilitate the ability to activate and select among multiple competing options according to the associative, multinetwork demands of semantic cognition. In contrast, we anticipated that modal controllability would facilitate the ability to retrieve specific exemplars from memory, perhaps facilitating cognitive associations when cues are weaker.

**Figure 1.**
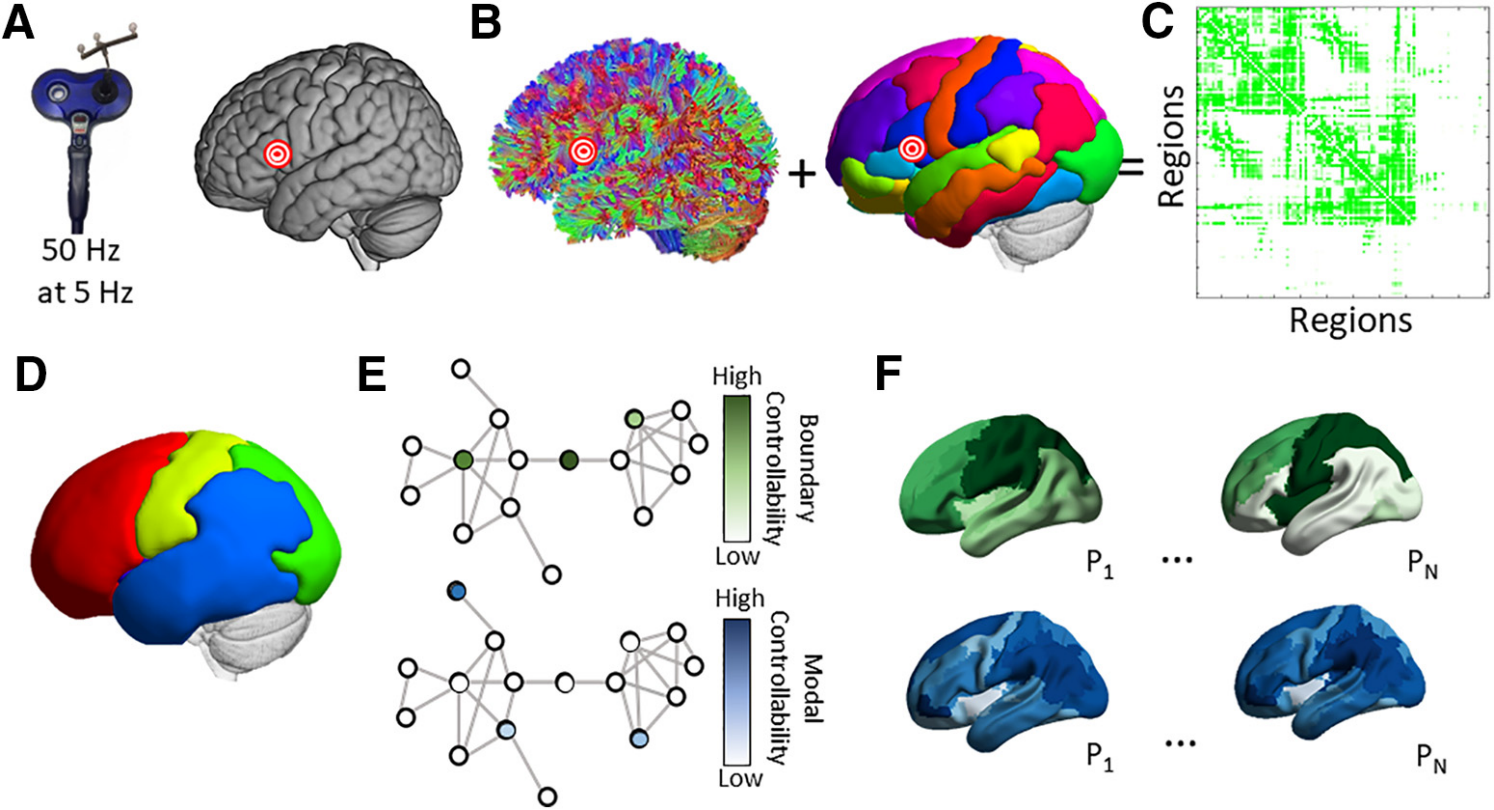
Overview of methods. ***A***, cTBS was administered to each subject’s pars triangularis (pictured with the bullseye) or the cranial vertex. ***B***, Diffusion tractography was computed for each subject. A cortical parcellation was registered to each individual’s anatomic T1 image to identify anatomic divisions. ***C***, A region × region anatomic adjacency matrix was constructed representing the streamline counts between pairs of regions corrected for region volume. ***D***, We applied a community detection algorithm to identify an initial consensus partition based on partitions identified within subjects. ***E***, Modal and boundary controllability were computed for each node (brain region) in the network for each individual. Each node received a rank representing its strength of control within the individual. ***F***, Maps representing the variability in modal controllability (top) and boundary controllability (bottom). *P*_1…_*_N_* represent different participants. The relationship between controllability values at the LIFG stimulation site and task RTs before and after stimulation were examined using mixed effects models.

To test our hypotheses, subjects participated in two experimental sessions (henceforth “pre-TMS” and “post-TMS”) in which subjects performed two language tasks with open-ended selection demands (verb generation and sentence completion) and one number naming task with a single appropriate response for comparison (not discussed here; see Medaglia et al., 2018a). Between the two task sessions, we administered either active or sham TMS. In the active TMS group, we administered continuous θ burst stimulation (cTBS), a form of TMS thought to induce neural inhibition for 60 min or more (Huang et al., 2005), to the pars triangularis within the LIFG. We chose this target given its role in generalized selection in semantic processing (Badre et al., 2005; Badre and Wagner, 2007), mediating cross-modal representation of spoken and written words (Liuzzi et al., 2017), and patient improvements in naming after inhibitory TMS to the right hemispheric homotope (Naeser et al., 2011; Harvey et al., 2017, 2019). In the sham TMS group, we administered TMS to the vertex in each subject. After the experiment was complete, we constructed anatomic brain networks from diffusion spectrum imaging (DSI) data acquired from each subject (Materials and Methods; Fig. 1*A*). Each network contained 111 brain regions defined by the Lausanne anatomic parcellation (Cammoun et al., 2012) and cerebellum (Diedrichsen et al., 2009; Fig. 1*B*), and each pair of regions was connected by an edge weighted by the number of streamlines linking those regions (Fig. 1*C*). We defined a simplified model of brain dynamics and simulated network control to quantify modal and boundary controllability (Fig. 1*D*).

### Neuroimaging: diffusion tractography

DSIs were acquired for all 41 subjects along with a T1-weighted anatomic scan at each scanning session. We followed a parallel strategy for data acquisition and construction of streamline adjacency matrices as in previous work applying network controllability statistics in human diffusion imaging networks (Gu et al., 2015; Betzel et al., 2016; Medaglia et al., 2018a). DSI scans sampled 257 directions using a Q5 half-shell acquisition scheme with a maximum *b* value of 5000 and an isotropic voxel size of 2.4 mm. We used an axial acquisition with the following parameters: repetition time (TR) = 5 s, echo time (TE) = 138 ms, 52 slices, field of view (FoV; 231, 231, 125 mm).

DSI data were eddy distortion corrected and reconstructed in DSI Studio (dsi-studio.labsolver.org) using *q*-space diffeomorphic reconstruction (QSDR; Yeh et al., 2011). QSDR first reconstructs diffusion-weighted images in native space and computes the quantitative anisotropy (QA) in each voxel. These QA values are used to warp the brain to a template QA volume in Montreal Neurologic Institute (MNI) space using a nonlinear registration algorithm. Once in MNI space, spin density functions were again reconstructed with a mean diffusion distance of 1.25 mm using three fiber orientations per voxel. Fiber tracking was performed in DSI Studio with an angular cutoff of 35°, step size of 1.0 mm, minimum length of 10 mm, spin density function smoothing of 0.0, maximum length of 400 mm and a QA threshold determined by DWI signal in the cerebrospinal fluid. Deterministic fiber tracking using a modified FACT algorithm was performed until 1,000,000 streamlines were reconstructed for each individual. DSI Studio placed starting points within seeding “voxels” at subvoxel resolution to account for potential partial volume influences on the fiber estimates (Campbell et al., 2005). The actual seeding points were determined randomly and uniformly within the voxels. DSI Studio used a deterministic random generator to place the seeds, and thus, the seeding sequence was both deterministic and random. These features ensured that the tracking result is reproducible using the same tracking parameters. DSI Studio drew a point within the voxel range using a uniform distribution. The point was then used as the starting point within the selected voxel.

Anatomical (T1) scans were segmented using FreeSurfer (Fischl, 2012) and parcellated using the connectome mapping toolkit (Cammoun et al., 2012) plus the Diedrichsen spatially unbiased cerebellum atlas (Diedrichsen et al., 2009). Compared with other functional parcellation schemes, our anatomic parcellation scheme ensures that we obtained networks from a consistent anatomic location within each subject, which is essential to supporting anatomic inferences and maintaining a consistent anatomic network location in each subject. The final parcellation scheme including *n *=* *111 regions was registered to the B0 volume from each subject’s DSI data. The B0 to MNI voxel mapping produced via QSDR was used to map region labels from native space to MNI coordinates. To extend region labels through the gray-white matter interface, the atlas was dilated by 4 mm (Cieslak and Grafton, 2014). Dilation was accomplished by filling non-labeled voxels with the statistical mode of their neighbors’ labels. In the event of a tie, one of the modes was arbitrarily selected. Each streamline was labeled according to its terminal region pair. From these data, we constructed a anatomic connectivity matrix, **A** whose element *A_ij_* represented the number of streamlines connecting different regions, divided by the sum of volumes for regions *i* and *j* (Hagmann et al., 2008). Notably, there are numerous free parameters in diffusion tractography, image parcellation, and graph representations of anatomic connectivity (e.g., weighted vs binarized or unweighted graphs).

### Cognitive testing

**Figure 2.**
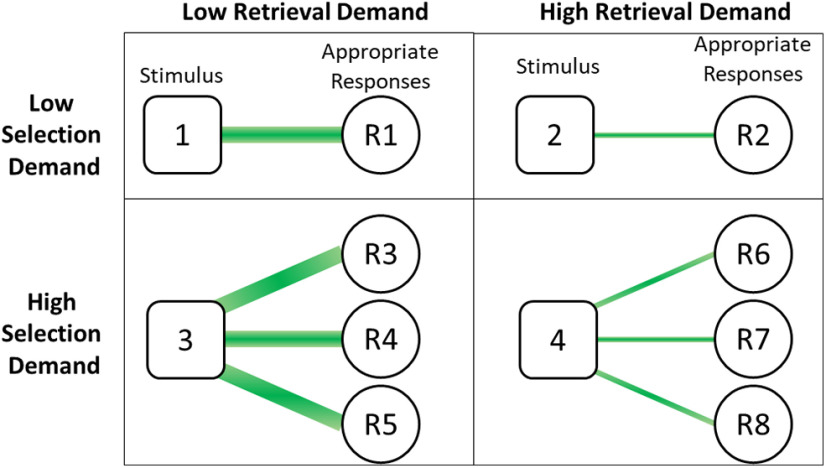
Selection and retrieval demands within the tasks. Items with high selection and low retrieval demands are those with many highly associated responses, and items with low selection and high retrieval demands are those with one weakly associated response. The stimuli were either verb cues in the verb generation task, or sentence cues in the sentence completion task. Even if selection and retrieval demands are similar in LSAs, each task places different predictive and syntactic demands on the semantic system that could influence performance. Selection and retrieval demands were measured continuously in a relative semantic space using LSA entropy and association strength, respectively, computed at the item level separately for each task.

Participants performed a verb generation and sentence completion task administered with ePrime 3.0 software on a desktop computer before and after receiving TMS (Snyder and Munakata, 2008; Snyder et al., 2014; Medaglia et al., 2018a; Fig. 2). All stimuli were written words presented on the screen in English. Subjects were asked to provide spoken responses to the tasks.

The order of tasks and order of task items (sentences/words) were counterbalanced across subjects, but within a subject’s session, the order of tasks remained the same pre-TMS versus post-TMS. Each task required ∼5 min. In addition, ∼5 min were required to set up and administer the cTBS sequence. Thus, the pre-TMS session (two language tasks), TMS administration, and post-TMS session (two language tasks) lasted a total of ∼25 min. Items (sentences/words) were not repeated within or between the sessions; half of the items per task were presented in the pre-TMS session and the other half were presented in the post-TMS session for a given subject. For the verb generation task, a single written word was presented on the screen, which remained on the screen for 10 s or until the participant made a response. For the sentence completion task, segments of one to two words were presented serially (1000 ms per segment) from left to right, starting with the beginning of the sentence. The sentences were presented accumulatively (the prior words remained on the screen until the response was given). Then, the whole sentence remained on the screen for 10 s starting from the onset of the final segment or until the participant made a response. The proportion of acceptable verb responses during the sentence completion task was low (12/100) and stratified across selection demands. For both tasks, trials were separated by the presentation of a fixation cross “+” for 500 ms. Subjects were given an example and five practice trials in the first administration of each language task (i.e., pre-TMS), and were reminded of the instructions before performing the task a second time (i.e., post-TMS). In each of the pre-TMS and post-TMS sessions, subjects completed 50 trials for a total of 100 trials per task.

For the verb generation task, subjects were instructed to generate the first verb that came to mind when presented with a noun stimulus (e.g., “cat”). The verb could be either something the noun does (e.g., “meow”) or something that is done with it (e.g., “feed”). Response times (RTs) were collected from the onset of the noun cue to the onset of the verb response. For the sentence completion task, participants were presented with a sentence, such as “They left the dirty dishes in the ––-.”, and were instructed to generate a single word that appropriately completes the sentence, such as “sink.” RTs were computed as the latency between the onset of the last segment, which always contained a two-word segment (i.e., a word and an underline), and the onset of the subject’s response. For both tasks, all items in the high versus low selection demand conditions were matched on retrieval demands (association strength; Snyder and Munakata, 2008).

The items for the verb generation task were identical to those used in Snyder et al. (2011) and the items for the sentence completion task were those from Snyder et al. (2014). The difficulty of items was sampled to cover a distribution of values computed via latent semantic analysis (LSA) applied to corpus data. In particular, items were sampled to represent a range of LSA entropy and LSA association strength (Snyder and Munakata, 2008), which represent the selection and retrieval demands of each item, respectively (Snyder and Munakata, 2008). An LSA association value of 0 means that the cue word or sentence is not strongly associated with any word in particular, whereas a value of 1 means that the cue word or sentence is strongly associated with at least one word, implying that it is easy to retrieve. An LSA entropy value of 0 indicates that the word is not related to any words, whereas higher values indicate higher relatedness to many words, which theoretically increases competition among appropriate words (Snyder and Munakata, 2008).

Verbal responses for all tasks were collected from a computer headset microphone. The microphone was calibrated to reduce sensitivity to environment background noise before the collection of data for each session such that the recording software was not triggered without clear verbalizations. List order was counterbalanced across participants and session (before or after active or sham stimulation). Item presentation order within each task was fully randomized across participants.

### TMS

The Brainsight system (Rogue Research) was used to co-register MRI data with the location of the subject and the TMS coil. The stimulation site was defined as the posterior extent of the pars triangularis in each individual subject’s registered T1 image. A Magstim Super Rapid^2^ Plus^1^ stimulator (Magstim) was used to deliver cTBS via a 70-mm diameter figure-eight coil. cTBS consisted of 50 Hz triplets administered every 200 ms (i.e., 5 Hz; Huang et al., 2005) for 600 total pulses. To calibrate the intensity of stimulation, cTBS was delivered at 80% of each participant’s active motor threshold (Huang et al., 2005). Each subject’s threshold was determined before the start of the experimental session using a standard up-down staircase procedure with stimulation to the motor cortex (M1). In the sham condition, the coil was held against the head at a 90° angle at the subject’s vertex to introduce a degree of induced electrical stimulation of the scalp. We administered sham at vertex to reduce the possibility that subjects could see the orientation of the coil in the sham condition, as subjects were not naive to TMS.

### Network controllability

To study the ability of a certain brain region to influence other regions in arbitrary ways we adopt the control theoretic notion of controllability. Controllability of a dynamical system refers to the possibility of driving the state of a dynamical system to a specific target state by means of an external control input (Liu et al., 2011; Pasqualetti et al., 2014; Ruths and Ruths, 2014). In the current paper, we follow the procedures applied in (Gu et al., 2015; Medaglia et al., 2018a) and focus on two network controllability statistics: boundary and modal controllability. Consistent with prior studies, we note that these statistics use linear discrete time dynamics that approximate nonlinear effects in simulations (Muldoon et al., 2016; Tiberi et al., 2017).

### Mathematical models

#### NCT

All network controllability measures were computed in MATLAB. We follow previous applications of NCT in diffusion weighted imaging data as the basis for our examination of controllability and cognitive control. We briefly describe the mathematical basis for the approach taken here. For a full discussion of anatomic network controllability in the context of diffusion weighted imaging networks, see (Gu et al., 2015). For a full discussion of the mathematical basis for anatomic network controllability see (Liu et al., 2011; Pasqualetti et al., 2014; Ruths and Ruths, 2014). In contrast to traditional graph theory, NCT offers mechanistic predictors of network dynamics. Mechanistic models can provide rich tests of causal dynamics in the human connectome by explicitly including a dynamic model (Medaglia et al., 2015).

The controllability of a networked system can be examined by defining a network represented by the graph *G = (V,E)*, where *V* and *E* are the vertex (node, or here, brain region) and edge (connection, here anatomic streamline density) sets, respectively. Let *a_ij_* be the weight associated with the edge (*i*,*j*) ∈ *E*, and define the weighted adjacency matrix of *G* as *A* = [*a_ij_*], where *a_ij_* = 0 whenever (*i*,*j*) ∉ *E*. We associate a real numeric value (state) with each node, collect the node states into a vector (network state), and define the map *x*:*N*_≥0_ → *R^n^* to describe the evolution (network dynamics) of the network state over time. Using the observed network and node dynamics, NCT can theoretically examine how the anatomic network structure relates to the types of control that nodes can exert.

#### Dynamic model of neural processes

Following prior work, we define anatomic brain networks by subdividing the entire brain into anatomically distinct brain areas (network nodes) in a commonly used anatomic atlas (Hagmann et al., 2008). Consistent with prior work (Bassett et al., 2011; Hermundstad et al., 2013, 2014; Gu et al., 2015), we connect nodes by the number of white matter streamlines identified by a commonly used deterministic tractography algorithm (Bassett et al., 2011; Hermundstad et al., 2013, 2014; Gu et al., 2015; Betzel et al., 2016; Tang et al., 2017; Cornblath et al., 2018; Stiso et al., 2019; Medaglia et al., 2018b; for details on the tractography implementation, see Medaglia et al., 2018a). This procedure results in sparse, weighted, undirected anatomic brain networks for each subject. Properties of this network include high clustering, short path length, and strong modularity, consistent with prior studies of similar network data (Hagmann et al., 2008; Bassett et al., 2011). The definition of anatomic brain networks based on tractography data in humans follows from our primary hypothesis that control features of neural dynamics are in part determined by the anatomic organization of the white matter in the brain.

As a simplified estimate of controllability at the region of interest, we drew from intuitions applied in other work linking network anatomy and function. (Honey et al., 2009, 2010; Abdelnour et al., 2014). Although neural activity evolves through neural circuits as a collection of nonlinear dynamic processes, these prior studies have demonstrated that a significant amount of variance in neural dynamics as measured by resting state fMRI can be predicted from simplified linear models. Based on this literature, we employ a simplified noise-free linear discrete-time and time-invariant network model:

(1) x(t+1)=Ax(t) + Bu(t),

where **x**:*R*_≥0_ → *R^n^* describes the state (e.g., a measure of the electrical charge, oxygen level, or firing rate) of brain regions over time, and **A** ∈ *R^N^*^×^*^N^* is a symmetric and weighted adjacency matrix. In this case, we construct a weighted adjacency matrix whose elements indicate the number of white matter streamlines connecting two different brain regions, denoted here as *i* and *j*, and we stabilize this matrix by dividing by the mean edge weight. While the model used above is a discrete-time system, the controllability Gramian is statistically similar to that obtained in a continuous-time system (Gu et al., 2015).

The diagonal elements of the matrix **A** satisfy *A_ij_* = 0. The input matrix **B***_K_* identifies the control points *K* in the brain, where *K* = {*k*_1_,…,*k_m_*} and

(2) $$B_{K}\text{ = [}e_{k}{}_{{}_{\text{1}}}\ldots e_{k}{}_{{}_{m}}\text{],}$$

and *e_i_* denotes the *i*-th canonical vector of dimension *N*. The input **u**:*R*_≥0_ → *R^m^* denotes the control energy.

#### Boundary controllability

Boundary controllability, a metric developed in NCT, quantifies the role of a network node in controlling dynamics between modules in hierarchical modular networks (Pasqualetti et al., 2014). Boundary controllability identifies brain areas that can theoretically steer the system into states where different cognitive systems are either coupled or decoupled. A region’s boundary controllability describes its theoretical ability to regulate the extent to which it can drive major networks to increase or decrease communication with one another. High boundary controllers are conceptually akin to the “gatekeepers” of communication between major brain networks. Here, we applied a similar approach to that taken in (Gu et al., 2015; Medaglia et al., 2018a) to quantify boundary controllability in our diffusion tractography networks and associate controllability variability with cognitive performance. Specifically, we partition the brain into modules by maximizing the modularity quality function (Newman, 2006) using a Louvain-like (Blondel et al., 2008) locally greedy algorithm (Jutla et al., 2011). Because the modularity quality function has many near-degeneracies, we optimized the algorithm multiple (100) times (Good et al., 2010).

Our approach differed from (Medaglia et al., 2018a) to include (1) full, weighted streamline networks and (2) partitions estimated within individuals. Given that anatomic network topology can vary across subjects and is explicitly of interest in examining the relationship between brain network organization, TMS, and behavior, we applied a tiered strategy to obtain a consistent partition threshold. First, we obtained partitions in each of 100 optimizations per subject at each value of γ from 1.0 to 4.0 in increments of 0.1. Next, we obtained the mean z-Rand coefficient for each subject and obtained the mean across subjects. We observed that the peak z-Rand across the sample was observed at γ at 2.0 (mean z-Rand score = 74.06, SD = 3.8). We therefore used the consensus partition at γ = 2.0 obtained from optimizations within each subject for the remainder of the analysis in this study. High-ranking boundary controllers were identified as the highest-ranking set of boundary regions between modules, and the remaining boundary regions were found within modules in the network.

#### Modal controllability

Modal controllability refers to the ability of a node to control each evolutionary mode of a dynamical network (Hamdan and Nayfeh, 1989), and can be used to identify the least controllable theoretical state from a set of control nodes. Modal controllability is computed from the eigenvector matrix *V* = [*v_ij_*] of the network adjacency matrix **A**. By extension from the PBH test (Kailath, 1980), if the entry *v_ij_* is small, then the *j*-th mode is poorly controllable from node *i*. Following Pasqualetti et al. (2014), we define $\varphi_{i}=\sum_{j=1}^{N}(1-\lambda_{j}^{2}(A))v_{ij}^{2}$ as a scaled measure of the controllability of all *N* modes *λ*_1_(*A*),….,*λ_N_*(*A*) from the brain region *i*. Regions with high modal controllability are able to control all the dynamic configurations of the network, and hence to drive the dynamics toward hard-to-reach configurations. A hard-to-reach state is one that requires a high amount of energy to reach. In the case of human brain networks, many competing and cooperating dynamics occur over time. As a result, the high-energy states typically involve the activation of a few, specific regions in the network that would otherwise express many coactivation patterns. High modal controllers are conceptually akin to dynamic “specialists” driving specific, otherwise unachievable states. Intuitively, a modal controller could correspond to one that is specialized to activate a single or small set of regions in the network, potentially supporting a few specific computational processes at a single location in the brain.

### Statistical analysis: examining the relationship between controllability, cognition, and TMS effects

This was a mixed study design with between-subjects effects of stimulation condition (active or sham TMS) and LIFG controllability, and within-subjects effects of item and selection and retrieval demands. To account for the study design, analyses were conducted using multilevel modeling with maximum-likelihood estimation (Baayen et al., 2008) implemented in the lme4 v.1.1-9 (Bates et al., 2015) package of R version 3.2.1 (R Core Team, 2016). This technique allows classical regression analyses to be performed on repeated measures data by accounting for the non-independence of observations collected from each participant (i.e., multiple behavioral observations obtained during the language tasks), without resorting to computing separate regression equations for each subject (Lorch and Myers, 1990; Baayen, 2008; Baayen et al., 2008). Critically, multilevel modeling accounts for the variances of the conditions of interest across subjects when estimating fixed effects, which is appropriate because of the potentially different effects of TMS across subjects (Lüders et al., 1985; Hamada et al., 2013). Multilevel modeling also accounts for violations of the sphericity assumption by modeling heteroskedasticity in the data when necessary, improving statistical power over other methods commonly employed for analyzing repeated-measures data.

We excluded from analyses trials on which participants responded incorrectly (i.e., semantic and paraphasic errors, hesitations, false starts) and experimenter error/equipment failures (such as false triggers for voice recording), constituting a mean of 4.25% and 4.67% of all trials, respectively. In addition, responses of <200 or >10,000 ms were excluded. We excluded responses below 200 ms because they are likely impulsive errors rather than those that reflect fast cognitive selection and retrieval and oral motor onsets (Indefrey and Levelt, 2004). In addition, compared with closed-ended language tasks with a single appropriate response, longer windows ensure that we measure task-relevant responses. Higher selection and retrieval demands tend to increase the central tendency and tail of RTs (Snyder and Munakata, 2008; Snyder et al., 2014). In early piloting we found that subjects occasionally provided semantically relevant responses after an 8- to 9-s delay, and the 10-s cutoff allowed us to be inclusive of some of these slower responses. See Table 1 for total trial rejection percentages for each task, TMS session, and group.

**Table 1:** Total trial rejection percentages for each session, task, and group

Session	Task	Group	Trial rejection percentage
Pre-TMS	Sentence completion	Active	7.20
	Sentence completion	Sham	10.125
Pre-TMS	Verb generation	Active	13.44
	Verb generation	Sham	10.500
Post-TMS	Sentence completion	Active	3.36
	Sentence completion	Sham	4.500
Post-TMS	Verb generation	Active	8.48
	Verb generation	Sham	8.125

All tables report the model estimates and parameter significance tests using Satterthwaite’s approximation. All mixed effects models included a random intercept for trials nested within subjects. Significant *p* values are denoted by bold text. The dependent variable in all models is the log of RTs during the tasks. In all models, CI = 95% confidence interval for the fixed effects estimates.

RTs were log-transformed because of non-normal distribution of raw RTs. For interactions with task variables, we discretized association and entropy values with a median split before computing interactions. Association and entropy values were centered and left continuous for interactions with the continuous controllability values.

Our modeling strategy was designed to test whether we replicated a prior finding that boundary controllability moderated performance on the tasks when considered together (Medaglia et al., 2018a). Then, we tested whether LIFG controllability was linked to TMS effects (1) between-task differences that suggest overall influences of semantic processing demands or (2) the within task selection and retrieval demands. First, we tested whether LIFG boundary controllability moderated TMS effects when both tasks were examined together as observed in our prior study (Medaglia et al., 2018a) in this larger sample with a modified data processing stream (i.e., full, weighted adjacency matrices and partitions for boundary controllability computed within subjects).

Then, we tested whether selection and retrieval demands, i.e., those measured by entropy and association strength in LSAs (Snyder et al., 2011, 2014), induced the same effect across the sentence completion and verb generation tasks. This would determine whether task-level distinctions because of differences in overall semantic integration demands exist before neuromodulation. In our models, a selection cost was represented by the main effect of entropy on RTs: slowed RTs in items with higher selection demands (i.e., greater entropy). Likewise, a retrieval cost was represented in our models by the main effect of association strength on RTs: slowed RTs for items with higher retrieval demands (i.e., lower association strengths). To test whether these costs were moderated by controllability, we examined whether baseline selection and retrieval costs were moderated by LIFG boundary and modal controllability in each task. Next, we tested whether session effects in the sham group differed across the tasks to examine whether interference observed in Medaglia et al. (2018a) increased in both. This established an important test for whether TMS alleviates interference observed in successive runs of language production as we speculated previously (Medaglia et al., 2018a). After testing for session effects (i.e., pre-TMS vs post-TMS outcome) in the sham group that could imply influences of increasing semantic interference (as indicated by slowed RTs (Medaglia et al., 2018a), we tested whether cTBS affected RTs on each task. Then, we examined whether LIFG controllability moderated observed TMS effects for each task. This analysis allowed us to determine whether the TMS effect was to mitigate this accumulated interference. The random effects structure for all models included a random slope for trial order nested within subjects (Barr et al., 2013).

### Code and data availability

Code for controllability measures can be found at: https://github.com/johnmedaglia/eneuro_controllability/. Data are available on request.

## Results

**Figure 3.**
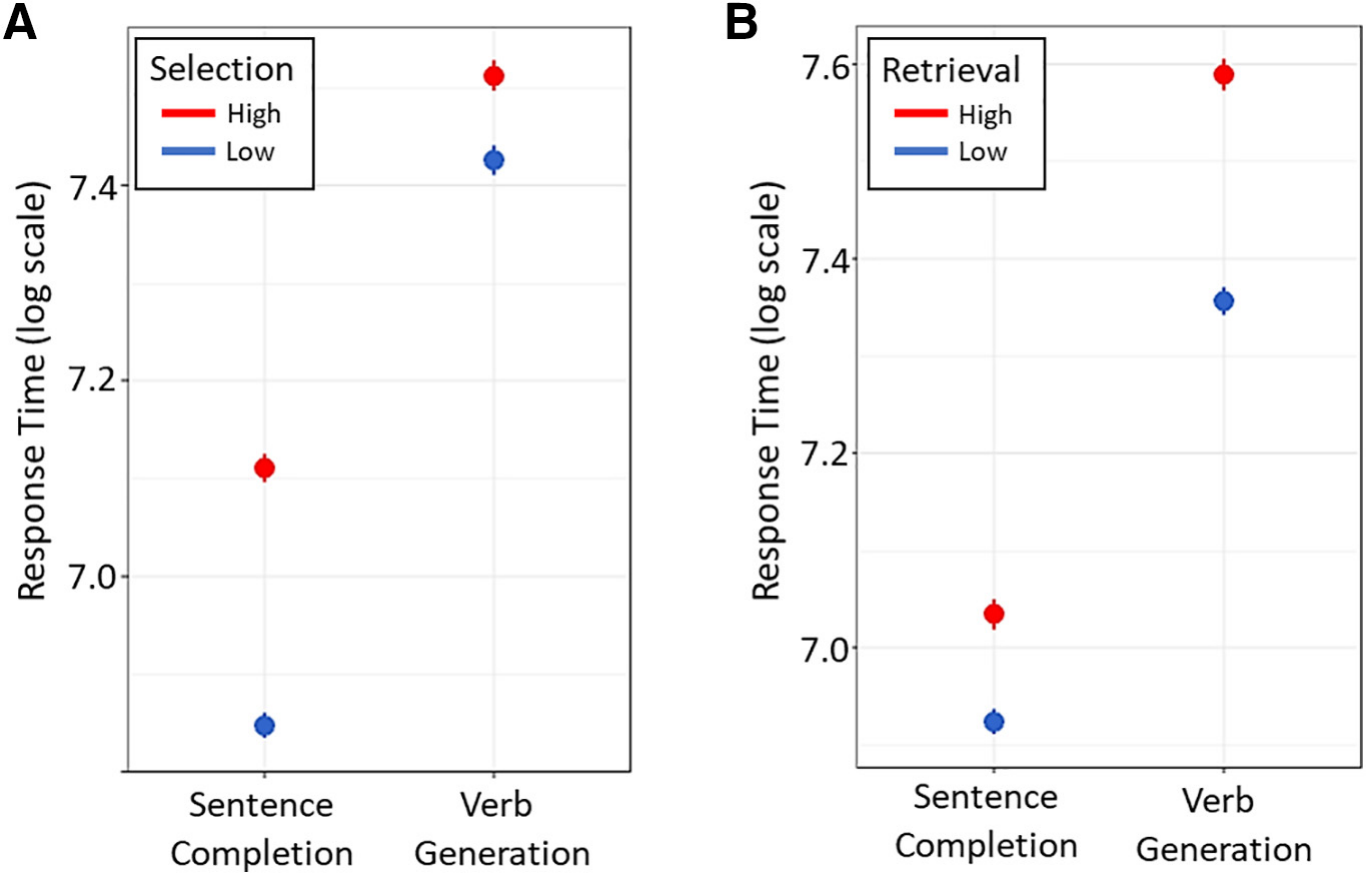
Selection and retrieval costs differ across language tasks. Selection costs were higher during the sentence completion task, whereas retrieval costs were higher in the verb generation task.

Across all sentence completion and verb generation data combined, we replicated the finding that LIFG boundary controllability was related to performance when both tasks were examined together (main effect of boundary controllability: β = −0.002, *p *=* *0.004; Table 2). In addition, boundary controllability moderated the TMS effect (stimulation × session × boundary controllability: β = 0.003, *p *=* *0.009; Table 2) In comparing the tasks, behavioral evidence revealed that the costs of these demands differed across the tasks overall before TMS. Selection costs (the effects of higher selection demands on performance) can be measured along a dimension as the parameter weight associated with item entropy values. Accordingly, retrieval costs (the effects of higher retrieval demands on performance) can be modeled as the parameter weight associated with item association strengths. Behavioral data revealed a task dissociation in pre-TMS selection and retrieval costs. Specifically, selection costs were greater in sentence completion (task by selection demand interaction: β = −0.180, *p *<* *0.001; Table 3), whereas retrieval costs were greater in verb generation (β = 0.122, *p *<* *0.001; Table 4). These differences suggest that differences in semantic demands exist at the task-level in addition to within-task variation in demands across items. See Figure 3 for estimated effects of selection and retrieval costs in the verb generation and sentence completion tasks pre-TMS.

**Table 2:** TMS effects depend on LIFG boundary controllability across both tasks

Predictors	Estimates	CI	Df	Statistic	*p*
(Intercept)	7.233	7.206 to 7.260	5609.031	522.049	**<0.001**
Stimulation	−0.030	−0.065 to 0.005	5608.513	−1.706	0.088
Session	0.024	−0.013 to 0.061	5770.483	1.256	0.209
Boundary	−0.002	−0.003 to −0.001	5675.063	−2.913	**0.004**
Stimulation × session	−0.035	−0.083 to 0.012	5775.291	−1.464	0.143
Stimulation × boundary	0.004	0.002 to 0.005	5650.508	5.440	**<0.001**
Session × boundary	0.001	−0.001 to 0.002	5824.119	0.718	0.473
Stimulation × session × boundary	−0.003	−0.004 to −0.001	5809.284	−2.629	**0.009**

The bold numbers indicate the statistically significant *p*-value of <0.05.

**Table 3:** Selection costs differ across the tasks at baseline

Predictors	Estimates	CI	df	Statistic	*p*
(Intercept)	6.846	6.818 to 6.874	3676.921	481.311	**<0.001**
Task	0.580	0.541 to 0.619	2899.285	29.214	**<0.001**
Selection	0.266	0.226 to 0.305	3638.267	13.177	**<0.001**
Task × selection	−0.180	−0.236 to −0.124	3615.092	−6.253	**<0.001**

The bold numbers indicate the statistically significant *p*-value of <0.05.

**Table 4:** Retrieval costs differ across the tasks at baseline

Predictors	Estimates	CI	df	Statistic	*P*
(Intercept)	6.925	6.897 to 6.952	3676.981	489.575	**<0.001**
Task	0.432	0.393 to 0.471	2893.463	21.924	**<0.001**
Retrieval	0.109	0.070 to 0.149	3644.480	5.394	**<0.001**
Task × retrieval	0.122	0.066 to 0.179	3628.856	4.228	**<0.001**

The bold numbers indicate the statistically significant *p*-value of <0.05.

**Figure 4.**
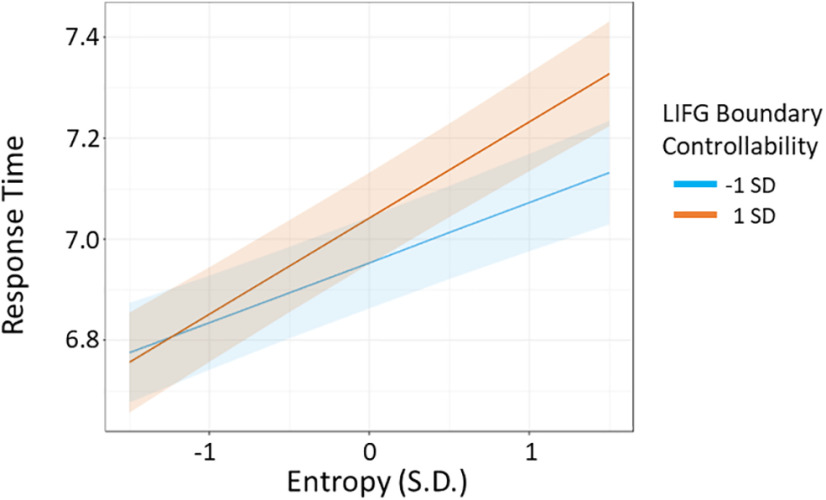
Boundary controllability moderates selection costs during sentence completion. Increased entropy values are associated with higher selection demands. A steeper positive slope of the relationship between entropy and RTs represents higher selection costs. Selection costs were higher at baseline in individuals with higher boundary controllability. To visualize the effects of the continuous boundary controllability values as a third dimension, we used a split of estimated regression lines from the models at −1 and 1 SDs of boundary controllability across the sample at baseline. For the exact model estimates for the main effects of entropy and LIFG boundary controllability and their interaction, see Table 5. SD, standard deviation.

After detecting task differences in selection and retrieval demands, we investigated whether LIFG network controllability moderated performance in response to cognitive demands at baseline. Following our behavioral data, we tested the link between LIFG boundary and modal controllability on (1) sentence completion and selection costs and (2) verb generation and retrieval costs. We found that the baseline selection costs were moderated by LIFG boundary controllability in sentence completion (LIFG boundary controllability by entropy interaction: β = 0.001, *p *=* *0.002; Table 5). The moderating influence of LIFG boundary controllability on the effects of entropy is illustrated in Figure 4. Modal controllability did not moderate selection demands during sentence completion (β = −0.006, *p *=* *0.063; Table 6). Neither boundary nor modal controllability significantly moderated baseline retrieval costs on verb generation (β = −0.001, *p *=* *0.587; Table 7; β = 0.003, *p *=* *0.702; Table 8).

**Table 5:** LIFG boundary controllability moderates baseline selection costs in sentence completion

Predictors	Estimates	CI	df	Statistic	*p*
(Intercept)	6.998	6.938 to 7.059	44.004	226.817	**<0.001**
Boundary	0.001	−0.001 to 0.004	40.167	1.284	0.199
Entropy	0.155	0.135 to 0.175	1833.751	15.293	**<0.001**
Boundary × entropy	0.001	0.000 to 0.002	1826.762	3.150	**0.002**

The bold numbers indicate the statistically significant *p*-value of <0.05.

**Table 6.** LIFG modal controllability does not moderate baseline selection costs in sentence completion

Predictors	Estimates	CI	df	Statistic	*p*
(Intercept)	7.422	7.353 to 7.490	53.769	211.851	**<0.001**
Modal	0.005	−0.004 to 0.013	42.251	1.106	0.269
Entropy	0.221	0.173 to 0.269	1757.626	8.990	**<0.001**
Modal × entropy	−0.006	−0.012 to 0.000	1736.954	−1.862	0.063

The bold numbers indicate the statistically significant *p*-value of <0.05.

**Table 7:** LIFG boundary controllability does not moderate baseline retrieval costs in verb generation

Predictors	Estimates	CI	df	Statistic	*p*
(Intercept)	7.521	7.453 to 7.588	52.950	217.976	**<0.001**
Boundary	0.001	−0.001 to 0.003	43.007	0.772	0.440
Association	−0.666	−0.774 to −0.557	1757.621	−12.029	**<0.001**
Boundary × association	−0.001	−0.005 to 0.003	1747.836	−0.543	0.587

The bold numbers indicate the statistically significant *p*-value of <0.05.

**Table 8:** LIFG modal controllability does not moderate retrieval costs in verb generation

Predictors	Estimates	CI	df	Statistic	*p*
(Intercept)	7.521	7.453 to 7.588	52.957	218.841	**<0.001**
Modal	0.003	−0.005 to 0.012	43.028	0.826	0.409
Association	−0.665	−0.773 to −0.556	1757.606	−12.018	**<0.001**
Modal × association	0.003	−0.011 to 0.017	1741.315	0.383	0.702

The bold numbers indicate the statistically significant *p*-value of <0.05.

**Figure 5.**
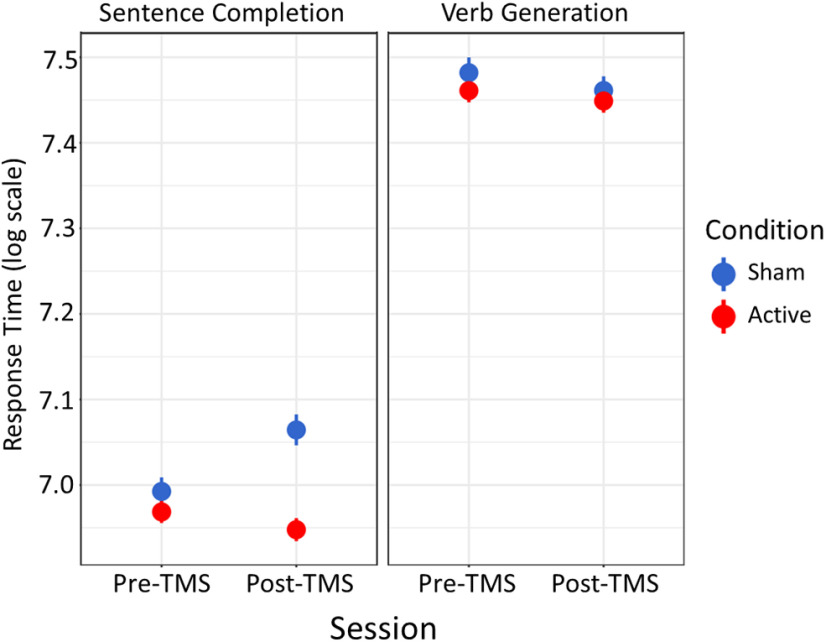
TMS Effects. In the sham group, responses on sentence completion slowed, whereas responses on verb generation slightly quickened. Inhibitory TMS improved sentence completion performance relative to sham.

In addition to differences in selection and retrieval costs across the tasks, we were interested in whether semantic interference in the sham group increased equally from the first to second session in each task. Differences across tasks could suggest that spreading activation causes increased competition in one task relative to the other with sustained task performance (Saunders and MacLeod, 2006; Nozari and Pinet, 2020). Session did not influence performance in both tasks: sentence completion RTs increased overall (β = 0.072, *p *=* *0.002) whereas verb generation did not (β = −0.022, *p *=* *0.319; Tables 9, 10). Thus, the increased context-driven nature of this task might induce more persistent, widespread activation of the semantic system that slows performance (Fig. 5, blue dots).

**Table 9:** Performance on sentence completion slows in the sham group across sessions

Predictors	Estimates	CI	df	Statistic	*p*
(Intercept)	6.993	6.959 to 7.027	1474.687	398.217	**<0.001**
Session	0.072	0.026 to 0.117	763.964	3.057	**0.002**

The bold numbers indicate the statistically significant *p*-value of <0.05.

**Table 10:** Performance on verb generation does not change in the sham group across sessions

Predictors	Estimates	CI	df	Statistic	*p*
(Intercept)	7.482	7.448 to 7.516	1409.983	432.892	**<0.001**
Session	−0.022	−0.064 to 0.021	744.236	−0.997	0.319

The bold numbers indicate the statistically significant *p*-value of <0.05.

**Figure 6.**
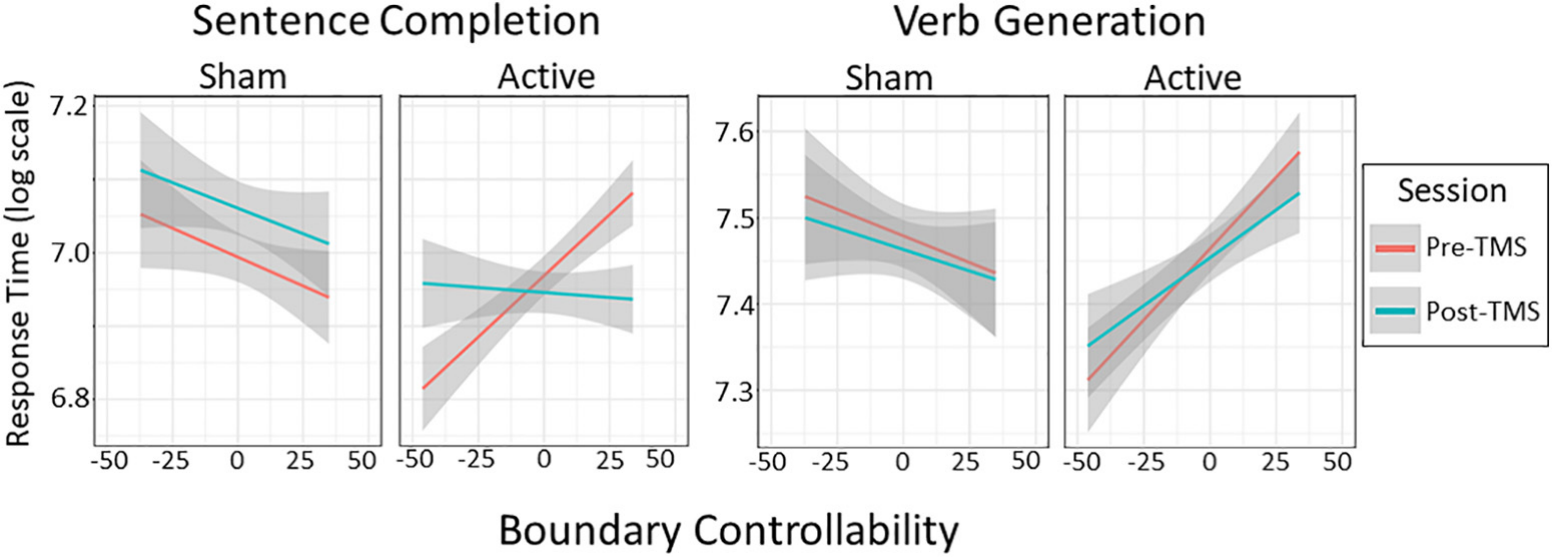
LIFG boundary controllability moderates TMS effects. TMS effects were moderated by LIFG boundary controllability specifically in sentence completion, where a crossover interaction was observed. Inhibitory TMS in individuals with higher boundary controllability attenuated the slowed performance observed pre-TMS among the active subjects. However, in verb generation, changes in RTs were consistently related to baseline performance in both the active and sham condition. Boundary controllability is plotted as the zero-centered rank controllability values at the LIFG across the sample. See Extended Data Figure 6-1 illustrating baseline differences in boundary controllability values between the active and sham groups. See Extended Data Figure 6-2 for a plot of all raw RT distributions by group, session, task, and selection and retrieval demands. See also Extended Data Figures 6-3 and 6-4 for trialwise modeling effects.

As illustrated in Figure 5, TMS influenced RTs only on sentence completion (stimulation by session interaction: β = −0.092, *p *=* *0.001; Table 11; stimulation by session interaction in verb generation: β = 0.009, *p *=* *0.750; Table 12), improving performance by removing the slowing effect observed in the sham group. Further dissociating the tasks, LIFG boundary controllability moderated the effect of inhibitory TMS only in sentence completion (LIFG boundary controllability by TMS by session interaction: β = −0.002, *p *=* *0.046; Table 13; verb generation: β = −0.002, *p *=* *0.146; Table 14). Thus, TMS effects were moderated by LIFG boundary controllability in the more semantically context-rich task. See Figure 6 for the estimated influence of boundary controllability on the TMS effect. Given the complex interaction, we conducted *post hoc* analyses of the boundary controllability values across individuals, finding that subjects in the active group had higher average boundary controllability values than those in the sham group (Wilcoxon unpaired two-samples ranked-sum test: *W *=* *1,776,167, *p *≪* *0.001; see Extended Data Fig. 6-1).

**Table 11:** TMS significantly speeds performance on sentence completion relative to the sham group

Predictors	Estimates	CI	df	Statistic	*p*
(Intercept)	6.993	6.960 to 7.027	3802.523	406.959	**<0.001**
Stimulation	−0.025	−0.068 to 0.018	3800.211	−1.139	0.255
Session	0.071	0.028 to 0.115	2000.452	3.204	**0.001**
Stimulation × session	−0.092	−0.148 to −0.036	1986.231	−3.245	**0.001**

The bold numbers indicate the statistically significant *p*-value of <0.05.

**Table 12:** TMS does not significantly affect performance on verb generation

Predictors	Estimates	CI	df	Statistic	*p*
(Intercept)	7.477	7.385 to 7.570	46.143	158.547	**<0.001**
Stimulation	−0.022	−0.141 to 0.096	46.311	−0.367	0.713
Session	−0.018	−0.062 to 0.025	3637.310	−0.829	0.407
Stimulation × session	0.009	−0.047 to 0.065	3637.744	0.318	0.750

The bold numbers indicate the statistically significant *p*-value of <0.05.

**Table 13:** LIFG boundary controllability moderates the TMS effect in sentence completion

Predictors	Estimates	CI	df	Statistic	*p*
(Intercept)	6.992	6.958 to 7.025	3799.047	407.782	**<0.001**
Stimulation	−0.025	−0.068 to 0.018	3797.234	−1.157	0.247
Session	0.070	0.027 to 0.114	1993.170	3.156	**0.002**
Boundary	−0.001	−0.003 to 0.000	3799.107	−1.837	0.066
Stimulation × session	−0.088	−0.144 to −0.033	1982.086	−3.123	**0.002**
Stimulation × boundary	0.003	0.002 to 0.005	3796.519	3.775	**<0.001**
Session × boundary	−0.000	−0.002 to 0.002	2019.562	−0.196	0.845
Stimulation × session × boundary	−0.002	−0.005 to −0.000	1996.384	−1.998	**0.046**

The bold numbers indicate the statistically significant *p*-value of <0.05.

**Table 14:** LIFG boundary controllability does not interact with TMS in verb generation

Predictors	Estimates	CI	df	Statistic	*p*
(Intercept)	7.478	7.445 to 7.512	3615.889	434.784	**<0.001**
Stimulation	−0.022	−0.065 to 0.021	3618.283	−0.993	0.321
Session	−0.019	−0.063 to 0.024	1894.733	−0.871	0.384
Boundary	−0.001	−0.003 to 0.000	3623.787	−1.571	0.116
Stimulation × session	0.010	−0.045 to 0.066	1902.848	0.368	0.713
Stimulation × boundary	0.003	0.001 to 0.005	3624.616	3.540	**<0.001**
Session × boundary	0.001	−0.001 to 0.002	1942.348	0.558	0.577
Stimulation × session × boundary	−0.002	−0.004 to 0.001	1940.439	−1.455	0.146

The bold numbers indicate the statistically significant *p*-value of <0.05.

10.1523/ENEURO.0382-20.2021.f6-1 Extended Data Figure 6-1 Boundary controllability differed between the active and sham groups. Subjects in the active stimulation condition had higher average values of boundary controllability. The upper and lower extents of the boxes represent the mean upper 75th percentile and lower 25th percentile of values, respectively. The whiskers represent the maximum and minimum range of values. Download Figure 6-1, TIF file.

10.1523/ENEURO.0382-20.2021.f6-2 Extended Data Figure 6-2 Raw RT distributions separated by task, TMS session, group, and selection and retrieval demands. Histograms represent the counts of RTs in each condition. Panels ***A–D*** in each subplot subdivide the data by median split along the selection (entropy) and retrieval (association strength) dimensions from the LSA analyses. Download Figure 6-2, TIF file.

10.1523/ENEURO.0382-20.2021.f6-3 Extended Data Figure 6-3 The influence of trial and selection demands on response times pre-TMS on the sentence completion task. Download Figure 6-3, DOC file.

10.1523/ENEURO.0382-20.2021.f6-4 Extended Data Figure 6-4 The influence of trial and selection demands on response times post-TMS. Download Figure 6-4, DOC file.

For further evaluation of whether accumulating interference or other temporal effects occurred during the tasks before and after TMS, we additionally explored trial-wise effects in the pre-TMS and post-TMS sentence completion data. Pre-TMS, subjects did not exhibit slowing overall (main effect of trial: β = 0.001, *p *=* *0.113), but greater slowing was observed among the items with higher selection demands (trial by selection interaction: β = 0.002, *p *=* *0.005). Post-TMS, subjects exhibited slowing overall (main effect of trial: β = 0.003, *p *=* *0.002), which was also greater among items with higher selection demands (trial by selection interaction: β = 0.002, *p *=* *0.001). See Extended Data Figure 6-2 for RT distributions for all conditions of the data. See also Extended Data Figures 6-3 and 6-4 for the complete modeling results for the trialwise pre-TMS and post-TMS effects.

## Discussion

We revealed novel associations between network controllability at the LIFG and controlled language functions. We found evidence linking boundary controllability to word selection and TMS effects during sentence completion. In partial agreement with our hypotheses, we revealed a link in the IFG between boundary controllability, the capacity for integrating and segregating activity across brain networks, and word selection in the context of the semantic demands of sentence processing. We did not find links between modal controllability and performance on either task or on selection and retrieval demands.

Consistent with theories that take a broad, whole-brain perspective on semantic processing (Patterson et al., 2007; Huth et al., 2012; Çukur et al., 2013; Bruffaerts et al., 2019; Shahdloo et al., 2020), part of the LIFG’s role in controlled language function could be to mediate the complex task of selecting context-dependent responses. In individuals whose LIFG is positioned to mediate between major brain networks (i.e., those with high LIFG boundary controllability), selection costs are increased. This suggests that as the LIFG increasingly mediates between brain networks, it is less able to either mitigate coactivation across semantic representations (Collins and Loftus, 1975; Anderson and Pirolli, 1984; Masson, 1995; De Deyne et al., 2016; Griffis et al., 2017; Mattheiss et al., 2018) or select among them (Canini et al., 2016; Beaty et al., 2017; Musz and Thompson-Schill, 2017; Abdel Rahman and Melinger, 2019). Moreover, task performance tends to slow on the second task administration in the sham group among individuals, especially on the sentence completion task. This effect could represent overall competition among representations increases over time on this task because of semantic priming. In addition, because higher boundary controllability indicates a stronger role in mediating inter-network communication, higher boundary controllability in the LIFG could imply that it is involved in managing additional demands in or outside the language domain (de Bruin et al., 2014). Although we cannot fully distinguish between the potential influences of fatigue or cognitive control in the absence of feedback and reward (Hockey, 2011; Dreisbach and Fischer, 2012; Shenhav et al., 2017), these possibilities could also explain part of the TMS effect that we observed.

Our results did not suggest a clear link between LIFG modal controllability and performance on either task or a relationship with either selection or retrieval demands. In anatomic brain networks, high modal controllability is strongly inversely related to node weighted degree (i.e., overall connectivity with nearest neighbors in the network; Gu et al., 2015). Thus, in persons with high LIFG modal controllability, the LIFG is more weakly connected with anatomic sites one step away in the network. These weaker connections may facilitate more limited, specific interactions with a few regions. This anatomic property might be especially relevant to retrieval demands when subjects attempt to recall single noun-verb pairs without the additional context provided by a complete sentence. For instance, when a noun is presented without context, it is potentially advantageous to interact with a smaller set of brain regions to increase the speed with which a simple association with an appropriate word can occur. This stands in contrast to the much richer semantic context required for sentence processing, which requires sequenced, persistent engagement of large set of brain networks to guide responses (Ni et al., 2000; Friederici, 2002; Cooke et al., 2006; Vigneau et al., 2006; Binder et al., 2009; Rogalsky and Hickok, 2009; Fedorenko and Thompson-Schill, 2014). In a prior study, modal controllability was only linked to performance on the closed-ended number reading task (Medaglia et al., 2018a). Thus, it is possible that modal controllability at the LIFG is restricted to cases without underdetermined competition, such as when only a single, well-associated exemplar (e.g., a number associated with a lexical form) is appropriate. If modal controllability is more generally linked to specific, well-learned representations, it is possible that it is more relevant to retrieving specific episodes and items with no competition.

Our TMS effects further provide evidence that LIFG boundary controllability moderates processing demands in language tasks with multiple processing demands. Pre-TMS, selection costs were more pronounced on sentence completion than verb generation and higher in those with stronger LIFG boundary controllability. Over sessions, slowed RTs occurred in the sham group only on sentence completion. Higher LIFG boundary controllability was associated with improved sentence completion performance after TMS. Thus, it is possible that the LIFG manages multinetwork processing demands. Stronger multinetwork anatomic connectivity could increase subjects’ proneness to semantic satiation (a transient loss of meaning) via repeated performance of the semantically rich sentence completion task. Further, inhibitory stimulation to the LIFG in individuals with higher boundary controllability might reduce more general demands on this region that are incurred by mediating among networks across the brain. For example, competition between the goal to stay on task versus attend to other tasks might further tax the LIFG in these individuals over time. Alternatively, domain-general cognitive control mechanisms could mediate slowed performance in the absence of reward, which is one basis of widely observed potential effort-reward tradeoffs in behavior (Shenhav et al., 2017), and a potential explanation of cognitive fatigue (Fukuda et al., 2010; Dobryakova et al., 2013; Milyavskaya et al., 2019). To test these possibilities, future studies could manipulate demands within and out of the language domain over several interleaved blocks of task performance. The role of reward on performance could be strong when high effort is predicted or required (Kool and Botvinick, 2014, 2018; Kool et al., 2017). Manipulating task demands and rewards in neuromodulation studies could further distinguish how variability in the network role of the LIFG mediates domain general and specific demands.

While our analyses focused on the anatomic connectivity of the LIFG, the mechanism of inhibitory TMS’s beneficial effect presumably involves local effects at the site of stimulation. Specifically, cTBS is thought to induce inhibition involving complex effects on GABAergic neurons (Gong et al., 2009; Stagg et al., 2009; Trippe et al., 2009; Cárdenas-Morales et al., 2010; Li et al., 2019). Previously, behavioral and computational work suggested that word selection can be facilitated using GABA agonists (Snyder et al., 2011). Our current findings point to the intriguing possibility that GABA-mediated mechanisms might parse the multinetwork demands on the LIFG. For instance, the LIFG’s ability to efficiently select task-relevant words might be especially challenged with sustained task effort when overall network demands on the LIFG are high. If the LIFG is inhibited (e.g., with TMS), the neural gains on task-relevant information in the network may be enhanced when the overall activity in this node is decreased (Houghton and Tipper, 1996; Ingham and McAlpine, 2005; Katzner et al., 2011), facilitating task-relevant responses (Houghton and Tipper, 1996; Herd et al., 2006). This benefit in healthy individuals could be linked to evidence in individuals with aphasia after stroke. Some individuals with aphasia benefit from inhibitory TMS to “noisy” node in the right inferior frontal gyrus, which sometimes inherits the role of the damaged LIFG poststroke (Torres et al., 2013). This notion could be examined by applying inhibitory stimulation to the right IFG poststroke in individuals with aphasia and observing whether language task performance improves.

More broadly, we note that the task demands and cognitive control in sentence completion and verb generation remain incompletely understood. Selection and retrieval demands might recruit anatomically different brain networks, which could explain the relative lack of findings linking retrieval to LIFG controllability. In addition, while we focused on the role of the LIFG with respect to the entire brain to be consistent with broad, whole-brain semantic theories, it is reasonable to suspect that classic theories of more specialized, left-lateralized language functions implicate a smaller set of networks to mediate these demands (Fedorenko, 2014). For example, circuits involving LIFG-anterior temporal lobe might be most relevant to selection (Musz and Thompson-Schill, 2017; Piai and Knight, 2018), while those involving the hippocampus might be more relevant to retrieval (Eldridge et al., 2000; Greenberg et al., 2005; Whitney et al., 2009). However, invasive neural recordings also suggest that these processes transiently recruit a wide swath of the cortex across the entire brain (Riès et al., 2017), challenging the assumption that a single-circuit model will be sufficient to account for these functions. Future studies could examine the role of single circuits and networks (Chai et al., 2016) with EEG and especially electrocorticography paired with anatomic diffusion tractography to obtain a more comprehensive, multinetwork model with good spatial and temporal resolution. Moreover, finer distinctions between domain-general and language domain-specific processes and regions could improve how we conceptualize task-level, selection, and retrieval demands (Ridderinkhof et al., 2004; Fedorenko, 2014; Fedorenko and Thompson-Schill, 2014; Blank and Fedorenko, 2017; Diachek et al., 2019). For instance, prior work applying TMS has dissociated semantic processing and phonological processing in the anterior and posterior LIFG, respectively (Hartwigsen et al., 2010; Ishkhanyan et al., 2020), with both contributing to grammatical sentence production (Hartwigsen et al., 2016). In addition, an important difference between the sentence completion and verb generation tasks is that sentences could be more likely to recruit predictive processes mediated through the LIFG (Altmann and Mirković, 2009; Arai and Keller, 2013; Yoshida et al., 2013; Grisoni et al., 2017; Vasishth et al., 2019), which we are not able to fully distinguish in the current study. Thus, investigating specific anatomic and functional pathways with tasks that dissociate these processes would further inform the relationship between LIFG anatomic connectivity and selection, retrieval, and other language production processes. Last and significantly, reward could be manipulated to dissociate task-related semantic satiation in the sentence completion task from reward-related processes (Shenhav et al., 2013; Kool and Botvinick, 2014, 2018; Kool et al., 2017).

Several limitations could be addressed with future studies. While our use of mixed effects modeling statistically accounts for unequal sample sizes and variances, the between-subject design and unequal samples are limitations. Future studies could use within-subjects crossover research designs with equal simple sizes. We used an anatomically-based approach to investigate the link between LIFG controllability and demands in controlled language performance. Here, our findings suggest that investigators should consider matching network measures of interest (controllability or others) across active and sham groups at the site of stimulation when feasible. As mentioned above, additional tasks that manipulate demand within and outside the language domain might further elucidate the relationship between the network control role of the LIFG and cognitive control. In addition, while we chose our anatomic network and tractography approach to be consistent with prior work using an anatomically-based atlas, diffusion tractography is fundamentally limited (Thomas et al., 2014; Maier-Hein et al., 2017) and other tractography and parcellation schemes are available. In particular, integrating well-established functional parcellations to focus on specific networks and their interactions could refine system-level predictions about the relationships between network controllability, language performance, and TMS-induced network effects (Beynel et al., 2020).

In our behavioral data, we also observed some pre-TMS differences across individuals with high and low boundary controllability in the active and sham groups. Most notably, boundary controllability was higher on average in the active group that was accompanied by an inversion in the model-estimated brain-behavior relationship in sentence completion pre-TMS. The TMS effect on this task appears to mitigate the slowing effect of boundary controllability on RTs in the active group subjects. In the current data, our results are unlikely to be accounted for by these pre-TMS differences. Our mixed effects modeling accounted for deviations in the active relative to the sham group. In the pre-TMS session, the relationship between boundary controllability and time was positive, meaning that subjects with higher boundary controllability were slower. Post-TMS, the relationship between boundary controllability and RTs was flattened. Thus, among individuals with relatively stronger boundary controllability in the LIFG, TMS could mitigate the influence of inter-network processing demands on average RTs during sentence completion. Nevertheless, it is clear that additional studies would be beneficial. Specifically, if sampling effects introduced pre-TMS differences at random, larger or prospectively assigned studies could obtain better matched pre-TMS for controllability or other network measures of interest. In addition, it is possible that other psychological differences that moderate controlled language functions such as anxiety could influence results (Snyder et al., 2014). Further, subjects responded to the verb generation task with verbs, whereas most responses to sentence completion were nouns. While we are unaware of specific prior data suggesting that the cognitive processes mediating spoken noun and verb production differ specifically with respect to the selection and retrieval demands studied here, this could be a topic for future studies. Moreover, our choice to stimulate pars triangularis might be more relevant to word selection than retrieval, and future studies could investigate whether controllability in the pars opercularis moderates performance in retrieval (Badre et al., 2005; Badre and Wagner, 2007). Lastly, the use of network controllability in diffusion tractography has several challenges. Questions remain about the appropriateness of linear approximations (Friston, 2008; Schiff, 2012; Gu et al., 2015), single-node control schemes (Tu et al., 2018; Pasqualetti et al., 2019; Suweis et al., 2019), and the relevance of network-wide estimations to processes involving local (cognitive) computations (Medaglia, 2019).

In conclusion, the emerging synergy between cognitive neuroscience and neural engineering provides many opportunities. Here, drawing from whole-brain theories of semantics, a potential link between the role of the LIFG in internetwork communication was examined with NCT. Overall, we found evidence that an increased role for the LIFG at the boundaries of major networks is potentially associated with resolving competition when processing sentences. This effect can be mitigated with inhibitory TMS in individuals whose LIFG serves a stronger role in inter-network connectivity. The mapping between general measures of node controllability and specific regional cognitive functions will require us to refine our models of cognitive control in language alongside our network imaging. Combining static anatomic measures with dynamic data (fMRI, EEG, electrocorticography) and neuromodulation could allow us to more specifically parse the distributed neural signals that mediate controlled language performance. In the long-term, refined models could allow us to enhance this critical human function in health and disease.

## Synthesis

Reviewing Editor: Macià Buades-Rotger, University of Luebeck Decisions are customarily a result of the Reviewing Editor and the peer reviewers coming together and discussing their recommendations until a consensus is reached. When revisions are invited, a fact-based synthesis statement explaining their decision and outlining what is needed to prepare a revision will be listed below. The following reviewer(s) agreed to reveal their identity: Hulya Aldemir, Jordi Martorell. Note: If this manuscript was transferred from JNeurosci and a decision was made to accept the manuscript without peer review, a brief statement to this effect will instead be what is listed below.

Dear authors,

Thank you for your thorough work concerning the issues raised in the first review round, which have substantially improved the readability of the manuscript. Nevertheless, there are still important issues to address. I detail these in the following:

Major comments:

- In the Abstract, the authors provide their predictions for both boundary and modal controllability. Right afterwards, they state their results only about boundary controllability, but nothing is said about modal controllability, which is a bit unexpected given the preceding sentences. Perhaps the authors could say something about the reported results both in favour and against their predictions.

- In the Significance statement, the authors state that “These findings provide a critical basis to reconcile local and connectionist models of language in the brain.”. Perhaps the label “connectionist” could be replaced by “whole-brain", as it is the one used (instead of “connectionist”) in the rest of the manuscript.

- The authors should specify which exact scripts they used to compute the controllability measures among the code available in the group’s website. They should also indicate the paper to which the scripts correspond. I assume one such script is modal_control.m from the Nat Com 2015 paper but neither the reviewers nor myself could identify the script for boundary controllability.

- The contribution of this manuscript relative to the previous J Neurosci article is still insufficiently justified. The authors should include brief sentence summarizing the Medaglia et al., 2018a results and detail how the present report complements or expands those results.

- In the Introduction, the authors added their predictions about boundary (line 132) and modal (line 135) controllability. However, these two terms - as well as the term “controllability” itself - have not been defined yet in the main text (only later in the methods section). Please provide a brief definition of these terms in order to make it easier to understand the logic of the predictions.

- In lines 246-249, the authors added that the “Stimulation sessions lasted approximately 15 minutes (5 minutes per task), with the two sessions occurring during the same experimental session separated by approximately 5 minutes (i.e., the time it took to set up and administer cTBS).” It is not clear to me how long the stimulation itself and both tasks in each session (pre- and post-TMS) lasted relative to each other according to this description. Please clarify it.

- In lines 253-254, the authors added how sentences were displayed. Although it could be somehow inferred from the following sentence, please mention explicitly that words were presented “accumulatively", as acknowledged in the rebuttal letter (“The sentences in the sentence completion task were presented accumulatively (the prior words remained on the screen until the response was given)”).

- In lines 261-262, the authors say “The verb could be either something the noun does (e.g., “meow”) or something you do with it (e.g., “feed”).”. This sentence could be easily written in a less informal way (e.g. “something that is done with it”).

- In the Methods sections, Boundary and modal controllability are well defined in their own terms and how they relate to brain-level dynamics. However, how they specifically relate to the two language tasks is not well developed (nor in the Introduction; see comment above)

- The results from Fig 4 based on standard deviations are still unexplained in the main text. As already noted in the former review comments, it is not clear from the main text how Fig 4 relates to Table 5. Please clarify it.

- Caption from Fig 5 does not say anything about the session effect on sham participants regarding both tasks.

- The explanation of TMS effects in both groups and tasks (lines 502-504) could make explicit reference to Fig 5 to facilitate the interpretation of these results to the reader.

- Caption of Fig 6 says “TMS effects were moderated by LIFG boundary controllability across both tasks.”. It seems that this is incorrect.

- The authors generally explain the results from Fig 6 as showing a facilitatory effect (active group, post-TMS, sentence task) for participants with higher boundary controllability. However, visual inspection of this figure suggests that the opposite is also true, as there is a detrimental effect (i.e., slower RTs) for participants with lower boundary controllability, resulting in the flat line when both effects are considered together. This issue is not mentioned in the text and it could be quite important, as it suggests other explanation not considered by the authors. For example, it could be that TMS eliminates the relationship between controllability and RTs (if there is any). Could the authors address this point?

- In lines 619-621, the authors added information about the potential role of predictive processing in the reported effects. Some reference to studies about predictive processing could be helpful.

Specific comments about rebuttal letter:

- The authors provide an additional plot (p. 4) to support that between-group differences before stimulation were not present. As the last Figure in the manuscript seems to suggest the opposite, it could be worth adding this additional information to the manuscript, and even consider adding the plot either in the manuscript or supplementary materials.

- The authors mention that, about the sentence task, “the proportion of acceptable verb responses was low (12/100) and stratified across selection demands.”. This could be added to the main text when describing the task.

- The authors provide a new figure (along with a statistical test in the text) showing that boundary controllability differed between groups (p. 13). It could be helpful to add this information to the manuscript, perhaps as supplementary material.

- The authors provide a new analysis showing trial effects (p. 15) pre-TMS. The authors should provide the same analysis for post-TMS data and include both in the manuscript, as these trial effects could either support or not the main arguments developed throughout the manuscript.

Minor comments:

- In key to the tables (p. 28), the levels of the session factor are still named as formerly.

- Typos: “difference” should be “different” in caption from Figure 2; something is missing in “the graph =(,), where and are the vertex...” (lines 314-315);

## Author Response

1

Thank you for your thorough work concerning the issues raised in the first review round, which

have substantially improved the readability of the manuscript. Nevertheless, there are still

important issues to address. I detail these in the following:

We appreciate the opportunity to address the reviewer’s additional comments. Our responses to

the critique are summarized below the reviewer’s specific comments (in italics) below.

Major comments:

- In the Abstract, the authors provide their predictions for both boundary and modal

controllability. Right afterwards, they state their results only about boundary controllability, but

nothing is said about modal controllability, which is a bit unexpected given the preceding

sentences. Perhaps the authors could say something about the reported results both in favour

and against their predictions.

We apologize for the omission. For completeness, have revised the Abstract to state:

"In contrast, modal controllability was not linked to performance on the tasks or TMS

effects.”

- In the Significance statement, the authors state that “These findings provide a critical basis to

reconcile local and connectionist models of language in the brain.”. Perhaps the label

"connectionist” could be replaced by “whole-brain", as it is the one used (instead of

"connectionist”) in the rest of the manuscript.

We agree and are sensitive to the broader implications of using the word “connectionist,” which

are not strictly evaluated here. We have applied this change.

- The authors should specify which exact scripts they used to compute the controllability

measures among the code available in the group’s website. They should also indicate the paper

to which the scripts correspond. I assume one such script is modal_control.m from the Nat Com

2015 paper but neither the reviewers nor myself could identify the script for boundary

controllability.

Thank you for noting this - we have now added the two scripts that compute modal

controllability and boundary controllability used here to our publicly available Github

corresponding to the analyses conducted in this paper. We now state in the Code and Data

Availability section:

"Code for controllability measures used specifically for the current study can be found at:

https://github.com/johnmedaglia/eneuro_controllability/"

- The contribution of this manuscript relative to the previous J Neurosci article is still 2

insufficiently justified. The authors should include brief sentence summarizing the Medaglia et

al., 2018a results and detail how the present report complements or expands those results.

Previously we had clarified that this manuscript focuses on the association between

controllability at the LIFG and performance on the different tasks as well as selection and

retrieval demands, but we also note that our current analyses used data from additional

subjects, focused on task-level distinctions, and refined our anatomical network construction.

We have added the following to page 4:

"Building on our previous study (Medaglia et al., 2018), the current study specifically

investigated (1) retrieval and selection demands in verbal language production (2) task-level differences in sentence completion and verb generation using weighted anatomical

networks.”

- In the Introduction, the authors added their predictions about boundary (line 132) and modal

(line 135) controllability. However, these two terms - as well as the term “controllability” itself -

have not been defined yet in the main text (only later in the methods section). Please provide a

brief definition of these terms to make it easier to understand the logic of the predictions.

Thank you for pointing this out. We have provided concise definitions for each of these terms

they first time they appear in the Introduction.

- In lines 246-249, the authors added that the “Stimulation sessions lasted approximately 15

minutes (5 minutes per task), with the two sessions occurring during the same experimental

session separated by approximately 5 minutes (i.e., the time it took to set up and administer

cTBS).” It is not clear to me how long the stimulation itself and both tasks in each session (pre- and post-TMS) lasted relative to each other according to this description. Please clarify it.

We apologize for the opaque description. We have revised this passage on page 9 to clarify the

total duration of a subject’s visit, the time per task before and after TMS, and the time required

to target and administer TMS:

"Each task required approximately 5 minutes. In addition, about 5 minutes were required

to set up and administer the cTBS sequence. Thus, the pre-TMS session (two language

tasks), TMS administration, and post-TMS session (two language tasks) lasted a total of

approximately 25 minutes.”

- In lines 253-254, the authors added how sentences were displayed. Although it could be

somehow inferred from the following sentence, please mention explicitly that words were

presented “accumulatively", as acknowledged in the rebuttal letter (“The sentences in the

sentence completion task were presented accumulatively (the prior words remained on the

screen until the response was given)”).

Thank you, we have revised this passage on page 9 to state:

"The sentences were presented accumulatively (the prior words remained on the screen

until the response was given). Then, the whole sentence remained on the screen for 10 3

seconds starting from the onset of the final segment or until the participant made a

response.”

- In lines 261-262, the authors say “The verb could be either something the noun does (e.g.,

"meow”) or something you do with it (e.g., “feed”).”. This sentence could be easily written in a

less informal way (e.g. “something that is done with it”).

Thank you, we have applied this change.

- In the Methods sections, Boundary and modal controllability are well defined in their own terms

and how they relate to brain-level dynamics. However, how they specifically relate to the two

language tasks is not well developed (nor in the Introduction; see comment above)

We believe that we are subject to journal space limitations in the Introduction that preclude a full

explication of the mapping between the theoretical and methodological details. However, we

have revised the Introduction as described above within those constraints. In addition, we have

used the Overview of Methods section to further explicate the ideas connecting controllability

measures, theoretical brain dynamics, and the language tasks on page 5 to state:

"Network controllability characterizes the theoretical ability of a node in a network (e.g., a

region in the brain) to drive the state of network activity (Liu et al., 2011). Here, we built

on our previous work linking boundary controllability to performance on open-ended

language tasks and modal controllability to closed-ended language tasks (Medaglia et

al., 2018). Specifically, the current study focused on task-level differences between two

open-ended tasks - sentence completion and verb generation - and two dimensions of

language demands - selection and retrieval (Snyder et al., 2008; Snyder et al., 2014).

Sentence completion task stimuli contain additional grammatical structure and

contextual semantics than verb generation task stimuli. Intuitively, we expected that

these processing demands would rely on multiple brain networks, and the theoretical

role of the LIFG in mediating among networks could be measured with boundary

controllability. In contrast, verb generation task stimuli might place greater demands on

the LIFG when subjects must obtain associations in the absence of additional task

structure or cues. We expected that if these demands are reflected in the LIFG’s role in

achieving difficult-to-reach states (i.e., specifically states of activation that are otherwise

difficult to activate in the network), we would find a relationship between performance on

verb generation and modal controllability. In addition, both tasks stratified selection and

retrieval demands at the item level, and we expected that the effects of these demands

on performance would be moderated by boundary and modal controllability, respectively.

We anticipated that boundary controllability would facilitate the ability to activate and

select among multiple competing options according to the associative, multi-network

demands of semantic cognition. In contrast, we anticipated that modal controllability

would facilitate the ability to retrieve specific exemplars from memory, perhaps

facilitating cognitive associations when cues are weaker.”

- The results from Fig 4 based on standard deviations are still unexplained in the main text. As 4

already noted in the former review comments, it is not clear from the main text how Fig 4 relates

to Table 5. Please clarify it.

We apologize for the confusion. In models where interaction terms are estimated using two

continuous main effects, the second variable is sometimes split at some interval to illustrate

effects. Here, the +/- 1 standard deviation split was used to more clearly visualize how boundary

controllability moderates the influence of selection demands (measured with LSA entropy) on

response times. We have modified the caption to make it clear how we have used the standard

deviation split for illustration, and that readers should refer to Table 5 for the exact model

estimates.

- Caption from Fig 5 does not say anything about the session effect on sham participants

regarding both tasks.

Thank you. We have added the following clarification to the Figure 5 caption:

"In the sham group, responses on sentence completion slowed, whereas responses on

verb generation slightly quickened.”

- The explanation of TMS effects in both groups and tasks (lines 502-504) could make explicit

reference to Fig 5 to facilitate the interpretation of these results to the reader.

Thank you, we now refer to Figure 5 in this passage.

- Caption of Fig 6 says “TMS effects were moderated by LIFG boundary controllability across

both tasks.”. It seems that this is incorrect.

We apologize for this oversight - we have corrected the caption to state:

"TMS effects were moderated by LIFG boundary controllability specifically in sentence

completion, where a crossover interaction was observed. Inhibitory TMS in individuals

with higher boundary controllability attenuated the slowed performance observed pre-TMS among the active subjects.”

- The authors generally explain the results from Fig 6 as showing a facilitatory effect (active

group, post-TMS, sentence task) for participants with higher boundary controllability. However,

visual inspection of this figure suggests that the opposite is also true, as there is a detrimental

effect (i.e., slower RTs) for participants with lower boundary controllability, resulting in the flat

line when both effects are considered together. This issue is not mentioned in the text and it

could be quite important, as it suggests other explanation not considered by the authors. For

example, it could be that TMS eliminates the relationship between controllability and RTs (if

there is any). Could the authors address this point?

We appreciate the reviewer further engaging with the interpretation of these findings as we

agree that it should be considered carefully. 5

In Fig. 6, the TMS effect was restricted to sentence completion. In the sham data, subjects in

the sham group with higher LIFG boundary controllability tended to be faster overall before and

after TMS.

In the active group, there was an opposite trend in the brain-behavior relationship before TMS,

where subjects with higher controllability tended to be slower. In the prior revision, we

suggested that this could be in part explained by baseline differences in group levels of

controllability that could be investigated in future larger studies with prospective group

assignments.

In the context of the active group, the TMS effect on this task appears to mitigate the slowing

effect of boundary controllability on RTs in the active group subjects. In the “Pre-TMS” session,

the relationship between boundary controllability and time was positive, meaning that subjects

with higher boundary controllability were slower. Post-TMS, the relationship between boundary

controllability and RTs was flattened. That effect suggests that in persons with this brain behavior relationship, TMS could mitigate the influence of inter-network processing demands on

responses during sentence completion. However, it is important if this effect is conditional on

the average level of controllability in the LIFG in the sample. To acknowledge this possibility

directly, we now state on page 21:

"Most notably, boundary controllability was higher on average in the active group that

was accompanied by an inversion in the model-estimated brain-behavior relationship in

sentence completion Pre-TMS. The TMS effect on this task appears to mitigate the

slowing effect of boundary controllability on RTs in the active group subjects. In the

current data, our results are unlikely to be accounted for by these pre-TMS differences.

Our mixed effects modeling accounted for deviations in the active relative to the sham

group. In the “Pre-TMS” session, the relationship between boundary controllability and

time was positive, meaning that subjects with higher boundary controllability were

slower. Post-TMS, the relationship between boundary controllability and RTs was

flattened. Thus, among individuals with relatively stronger boundary controllability in the

LIFG, TMS could mitigate the influence of inter-network processing demands on average

response times during sentence completion. Nevertheless, it is clear that additional

studies would be beneficial.”

- In lines 619-621, the authors added information about the potential role of predictive

processing in the reported effects. Some reference to studies about predictive processing could

be helpful.

Thank you, we have now added relevant cognitive, computational, and neural references that

elucidate some principles of predictive processes in language to the manuscript:

Altmann, G. T., & Mirković, J. (2009). Incrementality and prediction in human sentence

processing. Cognitive science, 33(4), 583-609.

Arai, M., & Keller, F. (2013). The use of verb-specific information for prediction in sentence

processing. Language and Cognitive Processes, 28(4), 525-560.

Grisoni, L., Miller, T. M., & Pulvermüller, F. (2017). Neural correlates of semantic prediction and

resolution in sentence processing. Journal of Neuroscience, 37(18), 4848-4858.6

Vasishth, S., Nicenboim, B., Engelmann, F., & Burchert, F. (2019). Computational models of

retrieval processes in sentence processing. Trends in cognitive sciences, 23(11), 968-982.

Yoshida, M., Dickey, M. W., & Sturt, P. (2013). Predictive processing of syntactic structure:

Sluicing and ellipsis in real-time sentence processing. Language and Cognitive Processes, 28(3),

272-302.

Specific comments about rebuttal letter:

- The authors provide an additional plot (p. 4) to support that between-group differences before

stimulation were not present. As the last Figure in the manuscript seems to suggest the

opposite, it could be worth adding this additional information to the manuscript, and even

consider adding the plot either in the manuscript or supplementary materials.

We agree that these plots are helpful to include. The median and distributions of the responses

were similar in the raw data illustrated in our first submission, whereas the estimated effects

over the range of boundary controllability values, sessions, & TMS conditions exhibited a more

complex interaction. We therefore believe that it is important to present the data in its raw form.

We have improved the figures from our first resubmission to journal-quality standard and

expanded them to include response time distributions by stimulation group, task, TMS session,

and level of selection and retrieval demands for comprehensiveness. We now reference these

data as Extended Data Figure 6-2 and refer to them in text and in the figure caption for Figure 6

in the main text.

- The authors mention that, about the sentence task, “the proportion of acceptable verb

responses was low (12/100) and stratified across selection demands.”. This could be added to

the main text when describing the task.

Thank you, we have added this to the description of the sentence completion task.

- The authors provide a new figure (along with a statistical test in the text) showing that

boundary controllability differed between groups (p. 13). It could be helpful to add this

information to the manuscript, perhaps as supplementary material.

We agree especially upon further consideration in response to our comments above with

respect to Figure 6. Consistent with eNeuro’s format we have included the figure representing

the boundary controllability values in the Active and Sham groups as Extended Data and refer

to them in the caption.

- The authors provide a new analysis showing trial effects (p. 15) pre-TMS. The authors should

provide the same analysis for post-TMS data and include both in the manuscript, as these trial

effects could either support or not the main arguments developed throughout the manuscript.7

We initially performed these tests for the pre-TMS data to aid interpretation of some of the brain behavior relationships at the trial level. However, we agree that the post-TMS analysis would be

of interest and that both results could be included in the manuscript.

Accordingly, we applied the same models used in the pre-TMS analysis to the post-TMS data.

Specifically, we tested the influence of trial and the interaction between trial and selection

demands on response times). Post-TMS, we found that the effect of trial was significant (β =

0.003, p = 0.002), as was the trial * selection interaction (β = 0.003, p < 0.001). This finding

suggested that accumulating interference was potentially evident post-TMS, whereas it was not

pre-TMS per our model in resubmission 1 (β = 0.001, p = 0.113). Consistent with the pre-TMS

data, the interaction term suggests that it is possible that there was additional accumulating

interference when subjects were challenged by items with high selection demands.

However, it remains justified to note that irrespective the trial-level analyses, the TMS

behavioral effect could potentially be explained by fatigue or unmanipulated cognitive control

demands in the absence of feedback or rewards. Those effects could cause subjects to

increasingly exhibit slowing performance across trials post-TMS relative to pre-TMS, as well as

continue to exhibit even greater slowing effects among items with high semantic interference.

That interpretation is reasonable because of the limitations of our experimental design that

cannot be fully resolved by the trial level analyses. Thus, we believe it is appropriate to share

the full results of these trial-level models but remain circumspect about the interpretation and

acknowledge them as a limitation requiring further study. We have added the modeling results

for the pre-TMS and post-TMS trial level analyses as Extended Data Tables 6-3 and 6-4 and

refer to them in the main text.

We have retained the note from our prior submission in the limitations section:

"Though we cannot fully distinguish between the potential influences of fatigue or

cognitive control in the absence of feedback and reward (Dreisbach et al., 2012, Hockey

et al., 2011; Shenhav et al., 2017), these possibilities could also explain part of the TMS

effect that we observed.”

As well as the statement:

"Alternatively, domain-general cognitive control mechanisms could mediate slowed

performance in the absence of reward, which is one basis of widely observed potential

effort-reward tradeoffs in behavior (Shenav et al., 2017), and a potential explanation of

cognitive fatigue (Dobryakova et al., 2013; Fukuda et al., 2010; Milyavskaya et al.,

2019).”

Minor comments:

- In key to the tables (p. 28), the levels of the session factor are still named as formerly.

Thank you, we have corrected the levels to be “Pre-TMS” and “Post-TMS”.

- Typos: “difference” should be “different” in caption from Figure 2; something is missing in “the

graph =(,), where and are the vertex...” (lines 314-315);8

Thank you, we have corrected these errors
