# Supplementary figures and images for: Language Tasks and the Network Control Role of the Left Inferior Frontal Gyrus
Source: eNeuro. 2021 Sep 8;8(5):ENEURO.0382-20.2021. doi: 10.1523/ENEURO.0382-20.2021 (PMC8431826; doi:10.1523/ENEURO.0382-20.2021)

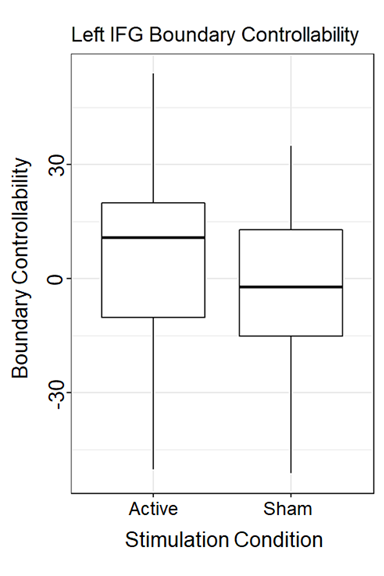

Supplement: Extended Data Figure 6-1 — Boundary controllability differed between the active and sham groups. Subjects in the active stimulation condition had higher average values of boundary controllability. The upper and lower extents of the boxes represent the mean upper 75th percentile and lower 25th percentile of values, respectively. The whiskers represent the maximum and minimum range of values. Download Figure 6-1, TIF file. [file enu-eN-CFN-0382-20-s01.tif]

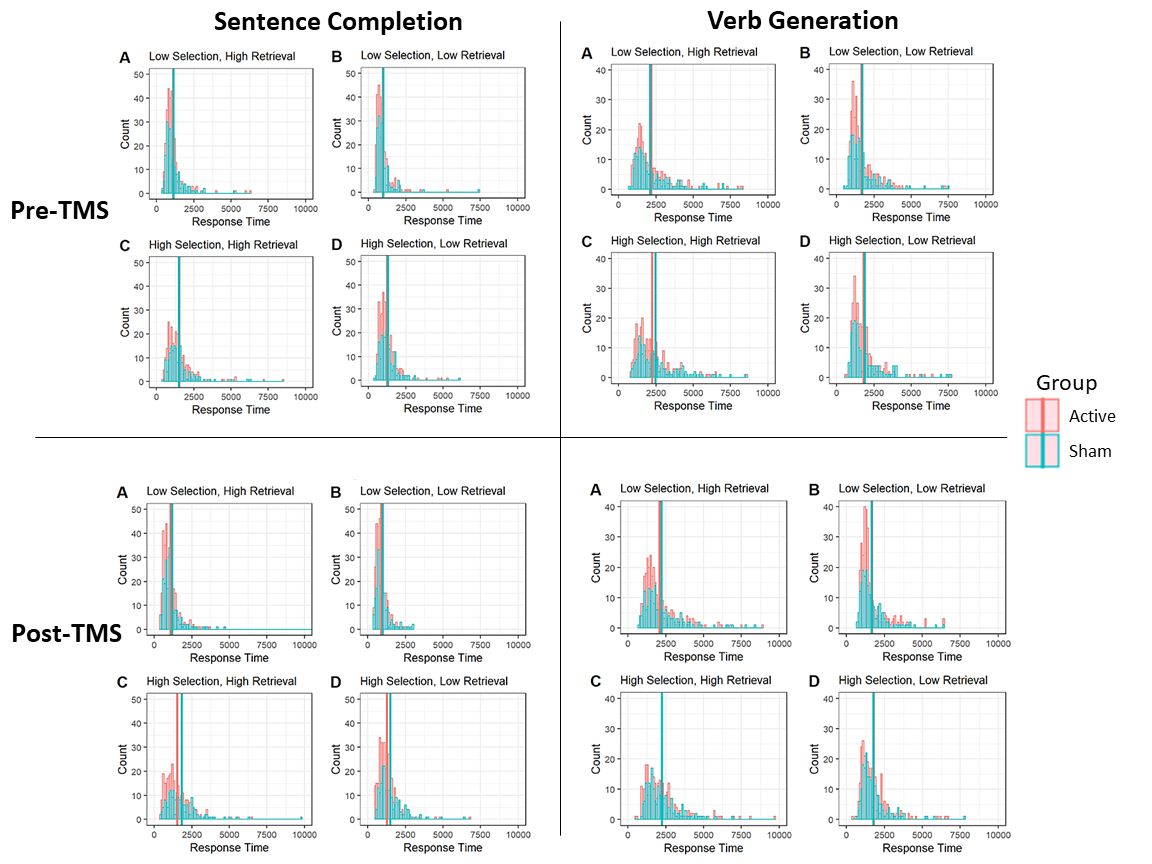

Supplement: Extended Data Figure 6-2 — Raw RT distributions separated by task, TMS session, group, and selection and retrieval demands. Histograms represent the counts of RTs in each condition. Panels A–D in each subplot subdivide the data by median split along the selection (entropy) and retrieval (association strength) dimensions from the LSA analyses. Download Figure 6-2, TIF file. [file enu-eN-CFN-0382-20-s02.tif]
