# Supplementary material for: Language Tasks and the Network Control Role of the Left Inferior Frontal Gyrus
Source: eNeuro. 2021 Sep 8;8(5):ENEURO.0382-20.2021. doi: 10.1523/ENEURO.0382-20.2021 (PMC8431826; doi:10.1523/ENEURO.0382-20.2021)
Supplement: Extended Data Figure 6-3 — The influence of trial and selection demands on response times pre-TMS on the sentence completion task. Download Figure 6-3, DOC file. [file enu-eN-CFN-0382-20-s03.doc]

# Figure 6-3: The influence of trial and selection demands on response times pre-TMS on the sentence completion task.

| *Predictors* | *Estimates* | *CI* | *df* | *Statistic* | *p* |
| --- | --- | --- | --- | --- | --- |
| (Intercept) | 7.233 | 7.223 – 7.243 | 1843 | 726.973 | **<0.001** |
| Trial | 0.001 | 0.000 – 0.002 | 38.94 | 1.621 | 0.113 |
| Selection | 0.131 | 0.121 – 0.141 | 5770.483 | 13.585 | **<0.001** |
| Trial * Selection | 0.002 | 0.001 – 0.003 | 5775.291 | 2.771 | **0.005** |
